# Utilization of diagnostic ultrasound and intravenous lipid-encapsulated perfluorocarbons in non-invasive targeted cardiovascular therapeutics

**DOI:** 10.1186/s40349-016-0062-y

**Published:** 2016-07-15

**Authors:** Thomas R. Porter, Songita A. Choudhury, Feng Xie

**Affiliations:** Department of Internal Medicine, Division of Cardiovascular Medicine, University of Nebraska Medical Center, 982265 Nebraska Medical Center, 68198 Omaha, NE USA

**Keywords:** Perfluorocarbons, Microbubbles, Diagnostic, Ultrasound, Targeted therapy

## Abstract

Diagnostic ultrasound (DUS) pressures have the ability to induce inertial cavitation (IC) of systemically administered microbubbles; this bioeffect has many diagnostic and therapeutic implications in cardiovascular care. Diagnostically, commercially available lipid-encapsulated perfluorocarbons (LEP) can be utilized to improve endocardial and vascular border delineation as well as assess myocardial perfusion. Therapeutically, the liquid jets induced by IC can alter endothelial function and dissolve thrombi within the immediate vicinity of the cavitating microbubbles. The cavitating LEP can also result in the localized release of any bound therapeutic substance at the site of insonation. DUS-induced IC has been tested in pre-clinical studies to determine what effect it has on acute vascular and microvascular thrombosis as well as nitric oxide (NO) release. These pre-clinical studies have consistently shown that DUS-induced IC of LEP is effective in restoring coronary vascular and microvascular flow in acute ST segment elevation myocardial infarction (STEMI), with microvascular flow improving even if upstream large vessel flow has not been achieved. The initial clinical trials examining the efficacy of short pulse duration DUS high mechanical index impulses in patients with STEMI are underway, and preliminary studies have suggested that earlier epicardial vessel recanalization can be achieved prior to arriving in the cardiac catheterization laboratory. DUS high mechanical index impulses have also been effective in pre-clinical studies for targeting DNA delivery that has restored islet cell function in type I diabetes and restored vascular flow in the extremities downstream from a peripheral vascular occlusion. Improvements in this technique will come from three dimensional arrays for therapeutic applications, more automated delivery techniques that can be applied in the field, and use of submicron-sized acoustically activated LEP droplets that may better permeate the clot prior to DUS activation and cavitation. This article will focus on these newer developments for DUS therapeutic applications.

**Electronic supplementary material:**

The online version of this article (doi:10.1186/s40349-016-0062-y) contains supplementary material, which is available to authorized users.

## Background

Although diagnostic ultrasound (DUS) systems and lipid-encapsulated perfluorocarbons (LEP) like Definity (Lantheus Medical) or Sonazoid (GE Healthcare) have been approved only for imaging applications; these two products have significant therapeutic potential for non-invasive targeted thrombolysis and drug delivery. Ultrasound and microbubbles alone as a method of dissolving thrombi was first introduced in 1997 [1] and was predicated upon work published just 1 year earlier by Tachibana and Tachibana demonstrating their potential to augment the effects of lytic therapy [2]. Subsequent in vivo studies demonstrated that ultrasound and microbubbles alone, using low-frequency non-imaging transducers, could recanalize peripheral vascular thrombi without fibrinolytic agents [3–5]. More recently, DUS pressures, despite their short pulse duration, have proven effective at recanalizing intravascular thrombotic occlusions [6]. The effectiveness was related to the use of intermittent high mechanical index impulses that are capable of causing both stable cavitation and inertial cavitation (IC). The intermittent application is necessary for microbubble permeation into the thrombus, and IC appears necessary, as this has been shown to create the fluid jets that erode thrombus both from outside and from within the thrombus infrastructure [6–8]. Subsequently, high mechanical index (MI) impulses from a DUS system have been used in both pre-clinical and clinical studies of acute ST segment elevation myocardial infarction (STEMI) and ischemic stroke, achieving successful coronary and cerebral recanalization with improved microvascular flow without the need of fibrinolytic therapy [9–14]. The DUS sequence has been modified slightly in each of these applications, but it is unclear how much of a modification beyond current diagnostic limits are required to achieve effective thrombolysis and targeted drug delivery. Numerous small animal studies have demonstrated the effectiveness of DUS-guided high MI impulses in targeting DNA [13–16], and more recent studies have demonstrated the potential of DUS to target the delivery of inhibitory RNA to suppress angiogenesis in adenocarcinoma [17, 18]. This review will focus on the data that has been accumulated regarding DUS efficacy in thrombolysis and drug delivery in large animals and how small modifications of current FDA-approved LEP may further improve their clinical potential.

## Review

### Targeted thrombolysis with DUS

**Fig. 1:**
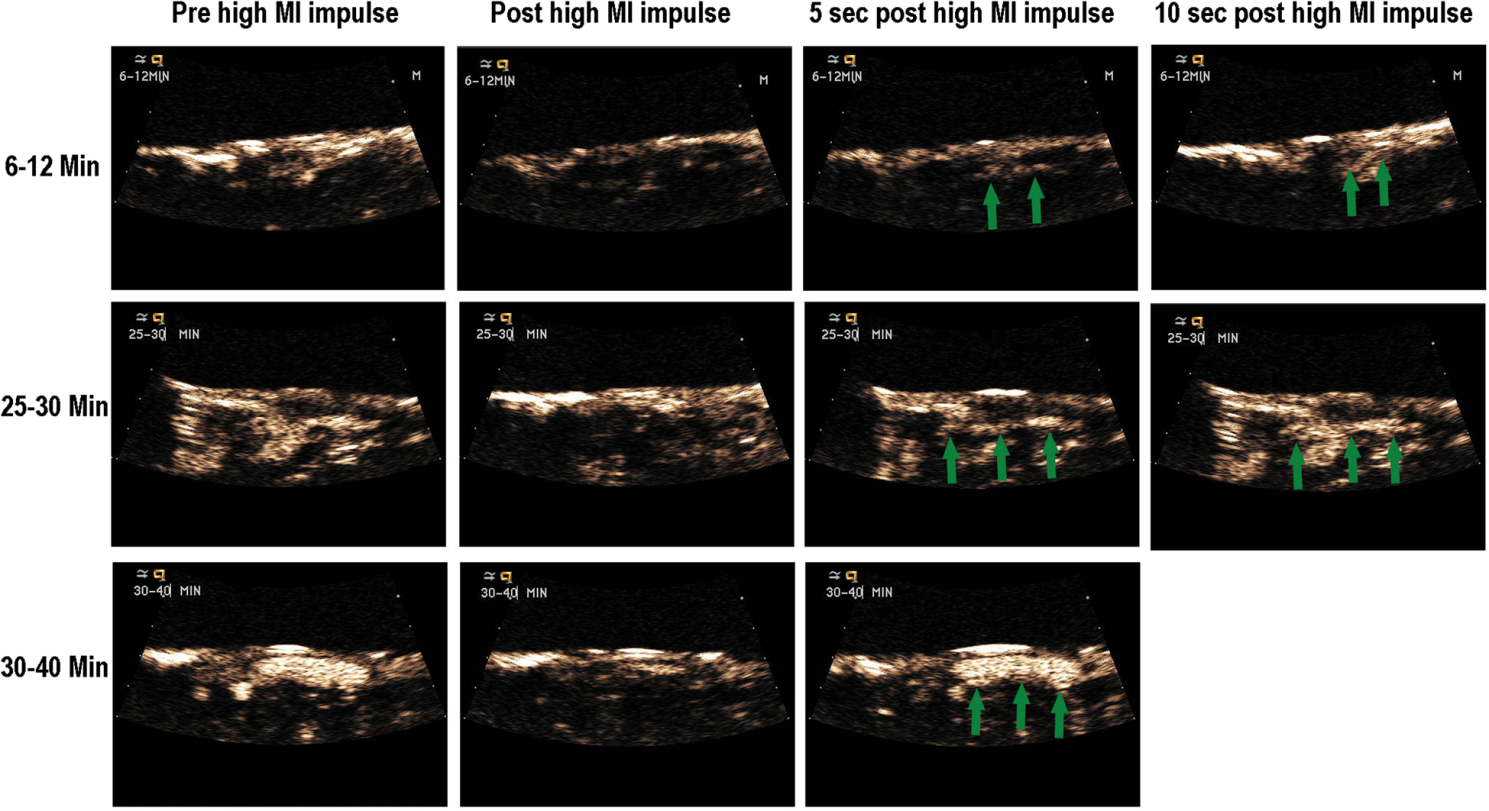
Very low MI images of a thrombosed arteriovenous graft when applying intermittent application of DUS high MI impulses during an intravenous infusion of LEP. Small channels that slowly replenish early in therapy (*top row* at 25–30 min of treatment) eventually become large channels that replenish more rapidly at 30–40 min of therapy. With permission

The potential for intermittent high MI impulses from a DUS transducer was first examined in a canine model of arteriovenous graft thrombosis, where intermittent high MI impulses (all <1.9 MI) were applied when low MI imaging detected microbubbles within the risk area [6]. These high MI impulses were shown to induce IC within the graft. The addition of the high MI impulses from a DUS transducer (Acuson Sequoia 512 1.5 MHz) through a 6-cm-thick tissue-mimicking phantom improved recanalization rates from 20 to 80 % after 30 min of treatment. What was interesting in this model is that although angiographically the vessel appeared to be occluded, low MI contrast-sensitive imaging with the DUS transducer detected microbubbles channeling through the thrombus before high MI impulses were applied (Fig. 1; Additional file 1). This presence of microbubbles was also used to guide when to apply high MI impulses; the small channels that were angiographically invisible at the start of guided high MI impulses progressed to larger channels with repeated high MI impulses (Fig. 1). Furthermore, these channels opened without any adjunctive fibrinolytic, anti-thrombotic, or anti-platelet agents, suggesting that mechanical thrombus dissolution was possible in the absence of pharmacotherapy. Furthermore, no significant downstream embolization was observed with ultrasound-induced recanalization.

**Fig. 2:**
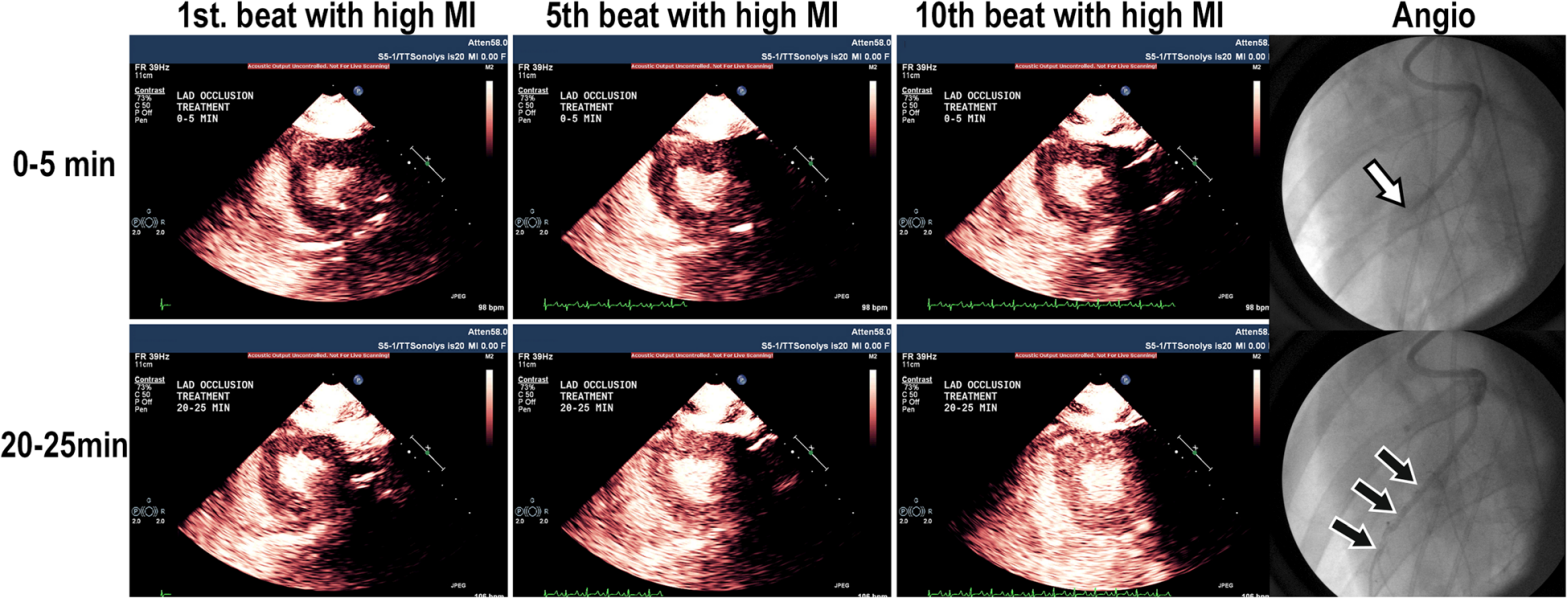
Parasternal short axis images and corresponding invasive angiograms of the left anterior descending during intermittent high MI applications of DUS while using very low MI imaging to examine the risk area in between high MI applications. At 20–25 min into therapy, the replenishment of myocardial contrast after a high MI application during the intravenous LEP microbubble infusion is more rapid and angiographic recanalization has occurred (*blue arrows*)

This study prompted subsequent investigations which examined the efficacy of DUS high MI impulses in restoring microvascular and epicardial blood flow in porcine models of acute ST segment elevation myocardial infarction, or STEMI [9–12]. These studies demonstrated that these same DUS high MI impulses were capable of restoring microvascular flow and function (Fig. 2; Additional file 2) even if epicardial recanalization was not achieved. Although epicardial recanalization rates tripled with the addition of platelet-targeted microbubbles and image-guided high MI DUS pulses, ST segment resolution (indicating microvascular recanalization) was seen with DUS high MI impulses even when epicardial recanalization was not observed, indicating that other potential mediators were playing a role in restoring microvascular flow. Subsequent studies in ischemic limb skeletal muscle downstream from a peripheral vessel ligation have confirmed that high MI DUS of LEP microbubbles can induce nitric oxide release, resulting in restoration of skeletal microvascular flow, despite persistent upstream vessel occlusion [15].

Slight prolongations in pulse duration on a DUS transducer may improve the amount of thrombus dissolution. By increasing pulse duration from <5 to 20 μs on a DUS transducer, a higher epicardial recanalization rate was achieved with DUS-guided therapy added to ½ dose tissue plasminogen activator. Despite the higher epicardial recanalization rate, both short and longer pulse duration high MI impulses were equally effective in ST segment resolution and improvement in wall thickening within the risk area [9]. The guided application of 20-μs pulse duration high MI impulses during an intravenous LEP microbubble infusion, without any fibrinolytic agent, was subsequently shown to produce equivalent epicardial recanalization rates as full dose fibrinolytic therapy in subsequent randomized comparisons in this same porcine model of acute STEMI [10]. Although this slight prolongation of pulse duration appears possible with current DUS transducers, the safety of this longer pulse duration has not been elucidated.

In an ischemic stroke, transcranial DUS and intravenous LEP microbubbles, even in the absence of tissue plasminogen activator, have recanalized intravascular and microvascular thrombi in a large porcine animal model [13]. In this model, the DUS was modified to provide an MI of 2.4 (pulse duration <5 μs, 1.7-MHz frequency) for guided therapeutic impulses, while low MI imaging was used to guide the applications. Other larger animal models have been utilized to determine the potential for transcranial ultrasound-induced cavitation of microbubbles to reduce stroke size [16], but without DUS pulse durations or imaging guidance. In humans, the initial studies using a pulsed wave Doppler (PWD) and systemically administered LEP in humans with acute ischemic stroke have all combined microbubbles with full dose fibrinolytic agents [17–19]. In the presence of systemically administered LEP, PWD was successful in increasing the speed of intracranial recanalization, but did also appear to increase the risk for intracranial hemorrhage. No human trials of ischemic stroke have examined the effect of targeted ultrasound-induced cavitation alone, without fibrinolytic agents. Such trials are needed and, based on acute STEMI studies, should be done with guided intermittent application of the high MI impulses. Table 1 lists the large animal studies that have utilized DUS-targeted IC to clear thrombi in specific clinical settings.

**Table 1:** DUS-guided cavitation with commercial available microbubbles: clinical applications in large animal models

Animal	Microbubbles	Transducer (MHz)	Application
Porcine	Lipid PFC	1.7	Catheter thrombi
Porcine	Lipid PFC	1.7	Graft hygiene
Porcine	Lipid PFC	1.7	Ischemic stroke
Human/porcine	Definity	1.7	STEMI

*PFC* perfluorocarbon

### Targeted drug and gene delivery with diagnostic ultrasound

**Fig. 3:**
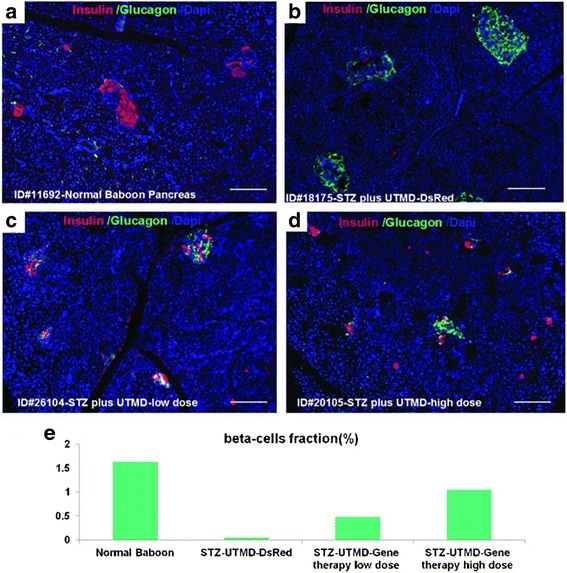
Demonstration of reformation of beta cells (*pink-stained cells*) following DUS-guided high MI-targeted delivery of plasmid DNA encoding for cyclin D2/CDK4/GLP1 in high doses (panel **d**). Panel **a** demonstrates a normal baboon pancreas. Note the numerous *pink-stained cells* that are now present. Panel **b** is UTMD control. Panel **c** is UTMD with low dose gene therapy. *Bottom panel* **e** demonstrates the fraction of beta cells present + US-guided therapies

A considerable number of small animal studies have demonstrated the ability of DUS-guided cavitation of LEP to target the delivery of DNA or RNA ([20–22]; Table 2). The delivery of DNA or short hairpin (sh) RNA can be achieved with binding of the nuclear material to cationic microbubbles. The cationic charge is achieved by altering the lipid composition of the microbubble shell. Current FDA-approved microbubbles like Definity (Lantheus Medical) do not have this charge and thus would not be expected to bind the negatively charged DNA or RNA [23]. There are few large animal studies demonstrating the potential for DUS in this area, but one group has now published preliminary studies for DUS-guided plasmid delivery of DNA (cyclin D2/CDK4/GLP1) in diabetic baboons to target the organized regeneration of beta cells into a functionally working pancreas (Fig. 3; Additional file 3). If this can be consistently demonstrated in a large non-human primate, the potential for safe targeted gene delivery with DUS and cationic LEP in humans should be evaluated [24, 25].

**Table 2:** Potential large animal application with DUS-targeted gene delivery

Disease entity	DUS frequency/trigging	Specific gene	Specific application
Diabetes	1:4	DNA-planned	Beta cell regeneration
Limb ischemia	1:4	DNA-planned VGE	Angiogenesis
Tumor		DNA	Tumor suppression
Tumor	1:10	ShRNA	Anti-angiogenesis

## Conclusions

The use of a commercially available LEP and DUS for targeted thrombolysis is now being tested in the first clinical trials [14], with promising initial results. Targeted IC of LEP has the potential not only to non-invasively and safely dissolve intravascular and microvascular thrombi but could also be effective in targeting gene delivery and has been demonstrated to target the delivery of vascular endothelial growth factors and genes for pancreatic regeneration. Diagnostic transducers have been modified in order to provide radiofrequency feedback to confirm IC has occurred, which may be necessary to confirm that a desired microbubble response has occurred [26].

One problem with microbubbles is that they are confined to intravascular spaces, and inertial cavitation can only increase subendothelial delivery. The LEP can also be formulated into droplets, even for the lower molecular weight fluorocarbons like octafluoropropane [27]. Since these droplets are nanometer scale, they can cross endothelial membranes and reach interstitial spaces, which may improve targeted delivery of genes into areas of myocardial scar, and improve thrombolysis efficacy by improving clot permeation prior to acoustic activation and inertial cavitation. Studies are ongoing now to explore this new potential for DUS and LEP.

## Abbreviations

DUS, diagnostic ultrasound; IC, inertial cavitation; LEP, lipid-encapsulated perfluorocarbons; MI, mechanical index; NO, nitric oxide; PWD, pulsed wave Doppler; STEMI, ST segment elevation myocardial infarction

## Additional files

Additional file 1: Consent for figure 1. (PDF 1214 kb) Additional file 2: Consent for figure 2. (PDF 152 kb) Additional file 3: Consent for figure 3. (PDF 1738 kb)
