# Supplementary material for: Utilization of diagnostic ultrasound and intravenous lipid-encapsulated perfluorocarbons in non-invasive targeted cardiovascular therapeutics
Source: J Ther Ultrasound. 2016 Jul 15;4:18. doi: 10.1186/s40349-016-0062-y (PMC4946285; doi:10.1186/s40349-016-0062-y)
Supplement: Additional file 1: — Consent for figure 1. (PDF 1214 kb) [file 40349_2016_62_MOESM1_ESM.pdf]

ELSEVIER LICENSE
TERMS AND CONDITIONS

Apr 29, 2016

This is a License Agreement between Feng Xie ("You") and Elsevier ("Elsevier") provided by Copyright Clearance Center ("CCC"). The license consists of your order details, the terms and conditions provided by Elsevier, and the payment terms and conditions.

All payments must be made in full to CCC. For payment instructions, please see information listed at the bottom of this form.

Supplier	Elsevier Limited<br>The Boulevard,Langford Lane<br>Kidlington,Oxford,OX5 1GB,UK
Registered Company Number	1982084
Customer name	Feng Xie
Customer address	Nebraska Medical Center
	Omaha, NE 68198
License number	3858271049554
License date	Apr 29, 2016
Licensed content publisher	Elsevier
Licensed content publication	JACC: Cardiovascular Imaging
Licensed content title	Treatment of Acute Intravascular Thrombi With Diagnostic Ultrasound and Intravenous Microbubbles
Licensed content author	Feng Xie,John Lof,Carr Everbach,Anming He,Richard M. Bennett,Terry Matsunaga,Jason Johanning,Thomas R. Porter
Licensed content date	April 2009
Licensed content volume number	2
Licensed content issue number	4
Number of pages	8
Start Page	511
End Page	518
Type of Use	reuse in a journal/magazine
Requestor type	author of new work
Intended publisher of new work	Springer Publishing Company
Portion	figures/tables/illustrations
Number of figures/tables/illustrations	1
Format	electronic
	Yes

Are you the author of this Elsevier article?	
Will you be translating?	No
Original figure numbers	Figure 5
Title of the article	Therapeutic Applications for Microbubbles
Publication new article is in	Current Cardiovascular Imaging Reports
Publisher of the new article	Springer Publishing Company
Author of new article	Porter TR, Xie F
Expected publication date	Jun 2016
Estimated size of new article (number of pages)	20
Elsevier VAT number	GB 494 6272 12
Permissions price	0.00 USD
VAT/Local Sales Tax	0.00 USD / 0.00 GBP
Total	0.00 USD

Terms and Conditions

INTRODUCTION

1. The publisher for this copyrighted material is Elsevier. By clicking "accept" in connection with completing this licensing transaction, you agree that the following terms and conditions apply to this transaction (along with the Billing and Payment terms and conditions established by Copyright Clearance Center, Inc. ("CCC"), at the time that you opened your Rightslink account and that are available at any time at http://myaccount.copyright.com).

GENERAL TERMS

2. Elsevier hereby grants you permission to reproduce the aforementioned material subject to the terms and conditions indicated.
3. Acknowledgement: If any part of the material to be used (for example, figures) has appeared in our publication with credit or acknowledgement to another source, permission must also be sought from that source. If such permission is not obtained then that material may not be included in your publication/copies. Suitable acknowledgement to the source must be made, either as a footnote or in a reference list at the end of your publication, as follows:
"Reprinted from Publication title, Vol /edition number, Author(s), Title of article / title of chapter, Pages No., Copyright (Year), with permission from Elsevier [OR APPLICABLE SOCIETY COPYRIGHT OWNER]." Also Lancet special credit - "Reprinted from The Lancet, Vol. number, Author(s), Title of article, Pages No., Copyright (Year), with permission from Elsevier."
4. Reproduction of this material is confined to the purpose and/or media for which permission is hereby given.
5. Altering/Modifying Material: Not Permitted. However figures and illustrations may be altered/adapted minimally to serve your work. Any other abbreviations, additions, deletions and/or any other alterations shall be made only with prior written authorization of Elsevier Ltd. (Please contact Elsevier at permissions@elsevier.com)
6. If the permission fee for the requested use of our material is waived in this instance, please be advised that your future requests for Elsevier materials may attract a fee.
