# Supplementary material for: Utilization of diagnostic ultrasound and intravenous lipid-encapsulated perfluorocarbons in non-invasive targeted cardiovascular therapeutics
Source: J Ther Ultrasound. 2016 Jul 15;4:18. doi: 10.1186/s40349-016-0062-y (PMC4946285; doi:10.1186/s40349-016-0062-y)
Supplement: Additional file 2: — Consent for figure 2. (PDF 152 kb) [file 40349_2016_62_MOESM2_ESM.pdf]

Gould, Carol A

From: plosone <plosone@plos.org>
Sent: Tuesday, November 03, 2015 2:38 PM
To: Gould, Carol A
Subject: RE: copyright permission [ref:_00DU0Ifis._500U0OoHQc:ref]

Dear Carol

Thank you for your message.

PLOS ONE publishes all of the content in the articles under an open access license called "CC-BY".

This license allows you to download, reuse, reprint, modify, distribute, and/or copy articles or images in PLOS journals, so long as the original creators are credited (e.g., including the article's citation and/or the image credit). Additional permissions are not required.

You can read about our open access license here:

http://www.plos.org/about/open-access/

There are many ways to access our content, including HTML, XML, and PDF versions of each article. Higher resolution versions of figures can be downloaded directly from the article.

Thank you for your interest in PLOS ONE and for your continued support of the Open Access model. Please do not hesitate to be in touch with any additional questions.

Kind Regards,

Davina Hodgkiss
Staff EO
PLOS ONE

Figure #2

--------------- Original Message ---------------
From: Gould, Carol A [carol.gould@unmc.edu]
Sent: 03/11/2015 16:04
To: plosone@plos.org
Subject: copyright permission

Good morning.

How do I go about getting copyright permission for a figure from an article that was published in PLOS One , July 2013, Vol 8/ issue 7. : Diagnostic Ultrasound induced inertial cavitiation to non-Invasively ... authors: Feng Xie, Shunji Gao, Juefei Wu...

Thank you
Carol A.Gould

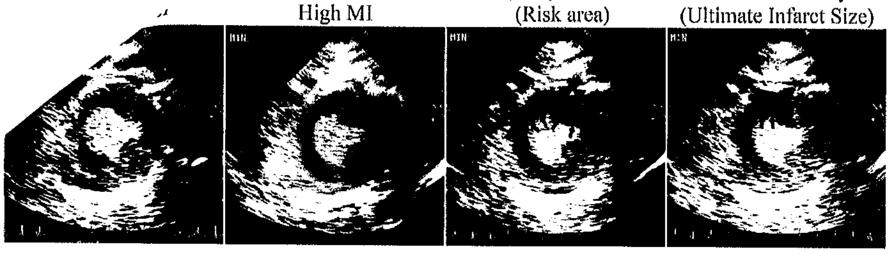

Figure 2. Myocardial Perfusion Images. Myocardial perfusion images from the low MI contrast imaging that were used to guide the app[illegible] of the therapeutic ultrasound (TUS) impulses. Note that after the TUS impulses, there was no myocardial contrast present in the left circumflex or left anterior descending perfusion beds (Second panel from the left). During low MI triggered imaging, early replenishment delineated the risk area (arrows; third panel from the left). A plateau intensity was reached (right panel), which was when the TUS impulses were re-applied.
doi:10.1371/journal.pone.0069780.g002

cavitational activity (which indicates the magnitude of growth and collapse of the microbubbles) between the 2.0 MI short pulse duration impulses and the 1.0 MI longer pulse duration impulses. However, since the 20 usec pulse lasts four times longer than the 5 usec pulse, the aggregate cavitational activity for the 20 usec pulse was nearly four times longer (0.73 volt-seconds versus 0.22 volt-seconds). These inertial cavitation signals were confirmed to be coming from the anterior myocardium, as they almost completely disappeared during LAD occlusion, and reappeared when applied following reperfusion of the LAD (Figure 4).

Hemodynamic Comparisons

Table 1 summarizes the hemodynamic, heart rate, and oxygen saturation measurements following coronary occlusion just prior to treatment, and at sixty minutes following initiation of treatment. There were no differences between groups in any of these parameters before or after randomized treatments, except activated clotting times due to heparin. The amount of thrombus required to occlude the LAD was 0.5±0.1 ml in Group I, 0.5±0.2 ml in Group II, and 0.5±0.2 ml in Group III. No ventricular arrhythmias were noted during application of either of the TUS impulses at any stage of the 30 minute treatment period.

Angiographic, Electrocardiographic, and Wall Thickening Measurements

The pigs in group III had an 83% angiographic recanalization rate at 30, 60 and 90 minutes (compared to 42%, 42% and 45% for Group I pigs or TPA alone, respectively; $p<0.05$). Recanalization rates for group II (short pulse TUS impulses) were 58% at these same time periods. Figure 3 is an example of a coronary angiogram of a recanalized left anterior descending artery after 30 minutes of treatment with the long duration 1.0 MI TUS impulse, as well as the improvement in myocardial perfusion within the risk area after treatment. In both Group II and III pigs, there was more rapid replenishment of microbubbles within the risk area following high MI impulses, as treatment time progressed (Figure 3).

Both short (Group II) and long (Group III) pulse TUS treated pigs exhibited improved microvascular flow by EKG. ST segment change improved 60% at 30 minutes into treatment in group III, compared to 48% in group II and 10% in group I ($P<0.05$ for both Group III and Group II *vs.* Group I). ST segment resolution >50% at 60 minutes into treatment occurred in 10 of 12 pigs (83%) in Group III, eight of 12 pigs (67%) in group II, and four of 12 pigs (33%) in Group I ($P = 0.006$). Wall thickening within the risk area at 60 minutes was significantly improved in Group III and Group II pigs compared to Group I.

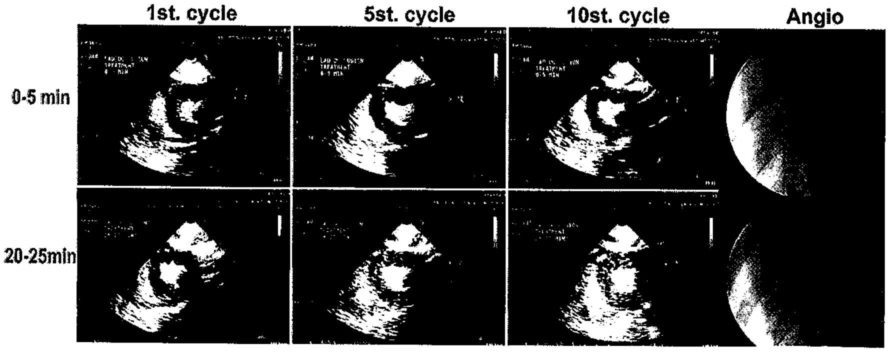

Figure 3. Intra-myocardial Passive Cavitation Detector Recordings During Therapeutic Impulses. Passive cavitation detector recordings from the anterior myocardium during a continuous infusion of microbubbles and application of the long pulse duration 1.0 MI (left panel) and short pulse 2.0 MI (right panel) therapeutic impulses. The black dots above the baseline signal represent inertial cavitational activity, which disappeared when the left anterior descending was occluded (The "TUS On" following balloon inflation), confirming that recorded activity was intra-myocardial. Note that the amplitude of the voltage, and number of recorded events produced by the cavitational spikes were similar for the two different therapeutic impulses. However, since the 20 usec pulse lasts four times longer than the 5 usec pulse, the aggregate cavitational activity for the 20 usec pulse is nearly four times longer (0.73 volt-seconds versus 0.22 volt-seconds).
doi:10.1371/journal.pone.0069780.g003
