# Supplementary material for: Utilization of diagnostic ultrasound and intravenous lipid-encapsulated perfluorocarbons in non-invasive targeted cardiovascular therapeutics
Source: J Ther Ultrasound. 2016 Jul 15;4:18. doi: 10.1186/s40349-016-0062-y (PMC4946285; doi:10.1186/s40349-016-0062-y)
Supplement: Additional file 3: — Consent for figure 3. (PDF 1738 kb) [file 40349_2016_62_MOESM3_ESM.pdf]

Copyright Clearance Center RightsLink®

Home | Account Info | Help | Live Chat

Taylor & Francis
Taylor & Francis Group
Journal Reprints

Title: Successful β cells islet regeneration in streptozotocin-induced diabetic baboons using ultrasound-targeted microbubble gene therapy with cyclinD2/CDK4/GLP1

Author: Shuyuan Chen, Raul A Bastarrachea, Brad J Roberts, et al

Publication: Cell Cycle

Publisher: Taylor & Francis

Date: Apr 1, 2014

Copyright © 2014 Taylor & Francis

Logged in as:
Feng Xie
Account #:
3000849608

LOGOUT

Review Order

Please review the order details and the associated terms and conditions.

Licensed Content Publisher	Taylor & Francis
Licensed Content Publication	Cell Cycle
Licensed Content Title	Successful β cells islet regeneration in streptozotocin-induced diabetic baboons using ultrasound-targeted microbubble gene therapy with cyclinD2/CDK4/GLP1
Licensed Content Author	Shuyuan Chen, Raul A Bastarrachea, Brad J Roberts, et al
Licensed Content Date	Apr 1, 2014
Licensed Content Volume	13
Licensed Content Issue	7
Type of Use	Journal/Magazine
Requestor type	academic/educational
Format	electronic
Portion	Figure/table/questionnaire
Number of figures/tables/questionnaires	1
Figure identification	Figure 1. Confocal microscopic images of insulin (red) glucagon (green) and Dapi (blue).
Will you be translating?	no
Circulation	50000
Does Taylor & Francis material represent more than 20% of your new work.	no
Author of this Taylor & Francis article	no
Order reference number	None
Title of the article	Utilization of diagnostic ultrasound and intravenous lipid encapsulated perfluorocarbons in Non-invasive targeted cardiovascular therapeutis
Publication the new article is in	Journal of Therapeutic Ultrasound
Publisher of the article	BioMed Central, the open access publisher
Author of new article	Thomas R. Porter, MD, Songita Choudhury,BS, Feng Xie, MD
Expected publication date	Feb 2016
Requestor Location	Feng Xie<br>Nebraska Medical Center<br>982265<br><br>Omaha, NE 68198<br>United States<br>Attn:
Total	145.20 USD

Copyright Clearance Center RightsLink®

Home | Create Account | Help | Live Chat

Taylor & Francis
Taylor & Francis Group
Journal Reprints

Title: Successful β cells islet regeneration in streptozotocin-induced diabetic baboons using ultrasound-targeted microbubble gene therapy with cyclinD2/CDK4/GLP1

Author: Shuyuan Chen, Raul A Bastarrachea, Brad J Roberts, et al

Publication: Cell Cycle

Publisher: Taylor & Francis

Date: Apr 1, 2014

Copyright © 2014 Taylor & Francis

LOGIN

If you're a copyright.com user, you can login to RightsLink using your copyright.com credentials. Already **a RightsLink user** or want to learn more?

Quick Price Estimate

**I would like to...**	reuse in a journal/magazine
**I am a/an...**	academic/educational
**I would like to use...**	Figure/table/questionnaire
**Number of figures/tables**	1
**My format is...**	electronic
**Does Taylor & Francis material represent more than 20% of your new work.**	no
**Are you the author of the requested content?**	no
**Circulation**	50000
**I am translating...**	no
**My currency is...**	USD - $
**Quick Price**	145.20 USD

This service provides permission for reuse only.
If you do not have a copy of the article you are using, you may copy and paste the content and reuse according to the terms of your agreement. Please be advised that obtaining the content you license is a separate transaction not including RightsLink.

Once licensed, you may reuse Taylor & Francis content according to the terms of your license. You will not receive a copy of the content.

QUICK PRICE | CONTINUE

To request permission for a type of use not listed, please contact the publisher directly.

Copyright © 2015 Copyright Clearance Center, Inc. All Rights Reserved. Privacy statement. Terms and Conditions.
Comments? We would like to hear from you. E-mail us at customercare@copyright.com

Copyright Clearance Center RightsLink®

Home | Account Info | Help | Live Chat

Taylor & Francis
Taylor & Francis Group
Journal Reprints

Title: Successful β cells islet regeneration in streptozotocin-induced diabetic baboons using ultrasound-targeted microbubble gene therapy with cyclinD2/CDK4/GLP1

Author: Shuyuan Chen, Raul A Bastarrachea, Brad J Roberts, et al

Publication: Cell Cycle

Publisher: Taylor & Francis

Date: Apr 1, 2014

Copyright © 2014 Taylor & Francis

Logged in as:
Feng Xie
Account #:
3000849608

LOGOUT

Choose Payment Method

Order Total: 145.20 USD

Pay Now By Credit Card

	Card Type	Card No.	Cardholder's Name	Exp. Date		
◉	Visa	***-2565	Carol Gould	09/2017	Change	Delete

Add new card

○ **Pay Later By Invoice**

Invoice Billing information

Customer Information (BILL TO)

**Name**	Feng Xie
**Address Line 1**	Nebraska Medical Center
**Address Line 2**	982265
**Address Line 3**	None
**City**	Omaha
**State/Province**	NE
**Zip/Postal Code**	68198
**Country**	United States
**Attention**	Feng Xie
**Customer Reference Information**	

You have the option to enter additional information for your company's reporting purposes.

☐ **I am initiating this transaction on behalf of a company not listed above.**

Edit Billing Information
